# A comparative study between outcomes of an in‐person versus online introductory field course

**DOI:** 10.1002/ece3.7209

**Published:** 2021-02-08

**Authors:** Alexandra I. Race, Maria De Jesus, Roxanne S. Beltran, Erika S. Zavaleta

**Affiliations:** ^1^ Education University of California Santa Cruz CA USA; ^2^ Biological Sciences Florida State University Tallahassee FL USA; ^3^ Ecology and Evolutionary Biology University of California Santa Cruz CA USA

**Keywords:** COVID‐19, distance/online learning, field courses, outcomes, student

## Abstract

The COVID‐19 pandemic has disrupted many standard approaches to STEM education. Particularly impacted were field courses, which rely on specific natural spaces often accessed through shared vehicles. As in‐person field courses have been found to be particularly impactful for undergraduate student success in the sciences, we aimed to compare and understand what factors may have been lost or gained during the conversion of an introductory field course to an online format. Using a mixed methods approach comparing data from online and in‐person field‐course offerings, we found that while community building was lost in the online format, online participants reported increased self‐efficacy in research and observation skills and connection to their local space. The online field course additionally provided positive mental health breaks for students who described the time outside as a much‐needed respite. We maintain that through intentional design, online field courses can provide participants with similar outcomes to in‐person field courses.

## Graphical abstract

As in‐person field courses have been found to be particularly impactful for undergraduate student success in the sciences, we aimed to compare and understand what factors may have been lost and/or gained during the conversion of an introductory field course to an online format. Using a mixed methods approach comparing data from online and in‐person field course offerings, we found that while community building was lost in the online format, online participants reported increased self‐efficacy in research and observation skills and connection to their local space. We maintain that through intentional design, online field courses can provide participants with similar outcomes to in‐person field courses.

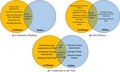

## 1 INTRODUCTION

Field courses provide students with valuable opportunities to learn physical and biological content and gain hands‐on practice in natural settings (Dillon et al., 2006; Durrant & Hartman, 2015; Easton & Gilburn, 2012; Hix, 2015; Morales et al., 2020). In their traditional in‐person formats, field courses are an important tool for retention, success, and equity in science majors, especially for underrepresented minorities (URM) (Beltran et al., 2020). These benefits emerge from the many positive factors impacted by field courses including self‐efficacy (Beltran et al., 2020; Dillon, 2013; Kortz et al., 2020), science and peer community (Epstein et al., 2015; Anderson and Miskimins, 2006; Haywood et al., 2016; Madden et al., 2012), and comfort in the outdoors (Carlone et al., 2016; van der Hoeven Kraft et al., 2011; Jolley et al., 2018). Despite these benefits, university support for field courses is diminishing, highlighting the importance of research that explores barriers, outcomes, and impacts of field courses (Cotton & Cotton, 2009; Moore, 2001; Smith, 2004).

The COVID‐19 pandemic shifted many standard approaches to science education. Nearly overnight, students and teachers were unable to access the classrooms, laboratories, and equipment necessary to promote hands‐on, inquiry‐driven learning. Teachers were challenged to convert instruction to online formats in meaningful ways, while students struggled to find structure and productive learning spaces in novel environments (Sahu, 2020). Laboratory and field courses were particularly impacted due to their dependence on specialized equipment and access to particular natural spaces in shared vehicles. Field‐course instructors worked creatively to restructure their field courses to an online format despite the concerns over reduced course outcomes (Barton, 2020). It is unclear whether this rapid conversion resulted in net gains or losses to students. On one hand, it is difficult to imagine how the development of skills, self‐efficacy, and community would be possible without in‐person interactions (Beltran et al., 2020; Carlone et al., 2016; Epstein et al., 2015; Mason et al., 2018). On the other, traditional field courses still remain inaccessible to many students, especially URM, due to course fees, institutional barriers (Cid & Bowser, 2015; Morales et al., 2020), limited sense of belonging (O’Brien et al., 2020; Smith et al., 2014), and physical disabilities that may be mitigated in an online course.

Our objective was to compare and understand which factors may have been lost and/or gained in the rapid conversion of field courses to an online format. We studied 201 students in in‐person and online offerings of a lower‐division field course, BIOE 82: Introduction to Field Research and Conservation, which is designed to minimize barriers for students interested in field experiences and to help students become familiar with the natural history of local ecosystems. While this course was able to maintain certain elements when transitioning online, such as field‐based observation, data collection, and a group research project, other aspects were not, like shared experiences outdoors and in‐person formation of relationships with instructors and peers during field research. To understand this impact, we compared data from reflective journal prompts, end of class focus groups, pre/post surveys, informal course evaluations, instructor interviews, and ethnographic case studies between in‐person and online offerings of *Intro to Field Research*.

## 2 METHODS

### 2.1 Setting

*Intro to Field Research* is a course taught at UC Santa Cruz designed to support frosh and transfer students' transition to college science courses, as it helps build student competence in practicing careful observations of nature, while also providing students with an understanding of how to implement the scientific process. The course is intentionally designed with features to maximize student access and success. These course features include small class size (Cuseo, 2007), group research projects (Alrefaie et al., 2020), curriculum vitae and cover letter support, course fee scholarships, and shared gear (Zavaleta, Beltran & Borker, 2020). To support students who may not feel comfortable in natural spaces, instructors carefully designed the course structure based on the mental, social, and physical factors that may help students feel at ease (hereafter, holistic support) (Fleischner et al., 2017).

Due to the global COVID‐19 pandemic, the course transitioned from in‐person to online in the Spring of 2020. The in‐person course structure included approximately eight lectures with four‐weekend field trips. The online course structure consisted of weekly Zoom lectures, quizzes, and frequent asynchronous work such as activities in their “Place in Space,” an outdoor location wherever they were in the United States. Both versions of the course had a group research project, with student interaction either in‐person (before the pandemic) or online (during the pandemic) (see Table A1).

### 2.2 Participants

There are no prerequisites for *Intro to Field Research,* so students from all majors are eligible to take the course. However, in the years this data were collected, the majority of course participants were intended biology majors. The demographics of this course vary across quarters but are generally reflective of the university, which enrolls approximately 30% URM (Latinx, Black, American Indian, and Pacific Islander) and 42% First Gen (see Table A2). Educational Opportunity Programs (EOP) participation is based on family income, undocumented, and foster care status. In‐person class size averaged around 23 and online averaged 14.

### 2.3 Data collection and analysis

This work was approved by UCSC IRB protocol #HS3230. Data for this study come from three quarters of in‐person instruction (Spring 2019, Fall 2019 and Winter 2020) and two quarters of online instruction (Spring 2020 and Summer 2020). Data collected included reflective journal prompts, end of class focus groups, pre/postsurveys, informal course evaluations, instructor interviews, and ethnographic case studies (see Table A3).

Data were collected using a mixed methods, case‐study approach. Each component of data collection had a set of questions that addressed different aspects of the course and field‐based learning experiences. The focus groups varied in numbers of participants (see Table A6). The questions included those pertaining to student motivations and aspirations and how field‐based courses played a role in those goals. We also focused on the barriers that students may have experienced in taking field‐based courses. In focus groups with only one student (focus group interview), additional topics included reflective prompts they had not previously answered, their experiences in the field, their expectations of the course, and course outcomes.

The pre‐ and post course surveys (responses in‐person pre = 271, post = 81; responses online pre = 33, post = 7) included Likert‐scale questions aimed to capture motivation, self‐efficacy, and community, and open‐ended questions about the students' expectations for the course as well as their understanding of conservation and environmental stewardship (see Table A6). Each student was asked to rate their confidence on a 5‐point Likert scale (1 = Strongly Disagree, 2 = Disagree, 3 = Neither Agree Nor Disagree, 4 = Agree, and 5 = Strongly Agree) for each of six questions: (a) I am familiar with the flora, fauna, and ecosystems of California; (b) I have strong experimental design skills; (c) I have strong oral presentation skills; (d) I know how to conduct field research projects from start to finish; (e) I am interested in pursuing a career in science; (f) I am interested in pursuing a graduate degree.

To provide a nuanced perspective on online learning settings, we also conducted interviews with one of the two online instructors and the teaching assistant (TA) from the online quarters. The questions focused on how they felt the online setting affected learning outcomes and student–teacher interactions.

We designed reflective journal prompts to capture changes in student self‐efficacy, community, and science identity (see Table A4). For example, one question stated “Did you do anything on this trip for the first time? What was it like? How do you feel now after the experience?”. We hoped this would organically elicit reflections on increased self‐efficacy as the students could talk about how they maneuvered learning a new skill. For the in‐person courses, we asked students to reflect on how different components of the trip impacted them through two questions per trip. With the transition to the online quarters, only two reflective prompts were implemented at the beginning and at the end of the course (note for the Spring Quarter reflective prompts were collected via the informal course evaluation). The number of responses varied between in‐person and online and the format collected (through google form or written in field notebooks) (see Table A3). The highest response rate (65%) occurred when responses were written in notebooks during the field trips in Fall 2019. The response rate to the online reflection prompts (average 15% across both online and in‐person quarters) was markedly lower (see Table A3). Though we cannot say why students were less likely to fill out online forms, we noticed there was less depth and profound reflection in the online responses compared to the written ones.

We conducted the case studies using ethnographic approaches including participant observation and field notes (Fall 2019 = 23 students observed; Summer 2020 = 15 students observed). These studies provided greater insights into the context and experience of the participants. Additionally, ethnographic approaches provided data on student discussion, connections, and experience that might not be captured in the reflective prompts.

To analyze our data, we created a codebook looking for themes of community, a sense of belonging, connection to scientific skills, and identity. We also were interested in holistic experiences students may have had in the course, looking to descriptions of emotions felt by students. We coded as a team to ensure consistency. After the codebook was finalized, we used the qualitative software Dedoose to aid in our analysis of the journal entries, interviews, focus groups, and open‐ended responses to the surveys. To triangulate our findings, we used the quantitative survey data and case‐study field notes. Due to the small sample sizes, the quantitative survey data were analyzed using a cross‐sectional approach (i.e., mean of postsurveys minus mean of presurveys for all students, including those that only answered the presurvey).

## 3 RESULTS

**FIGURE 1:**
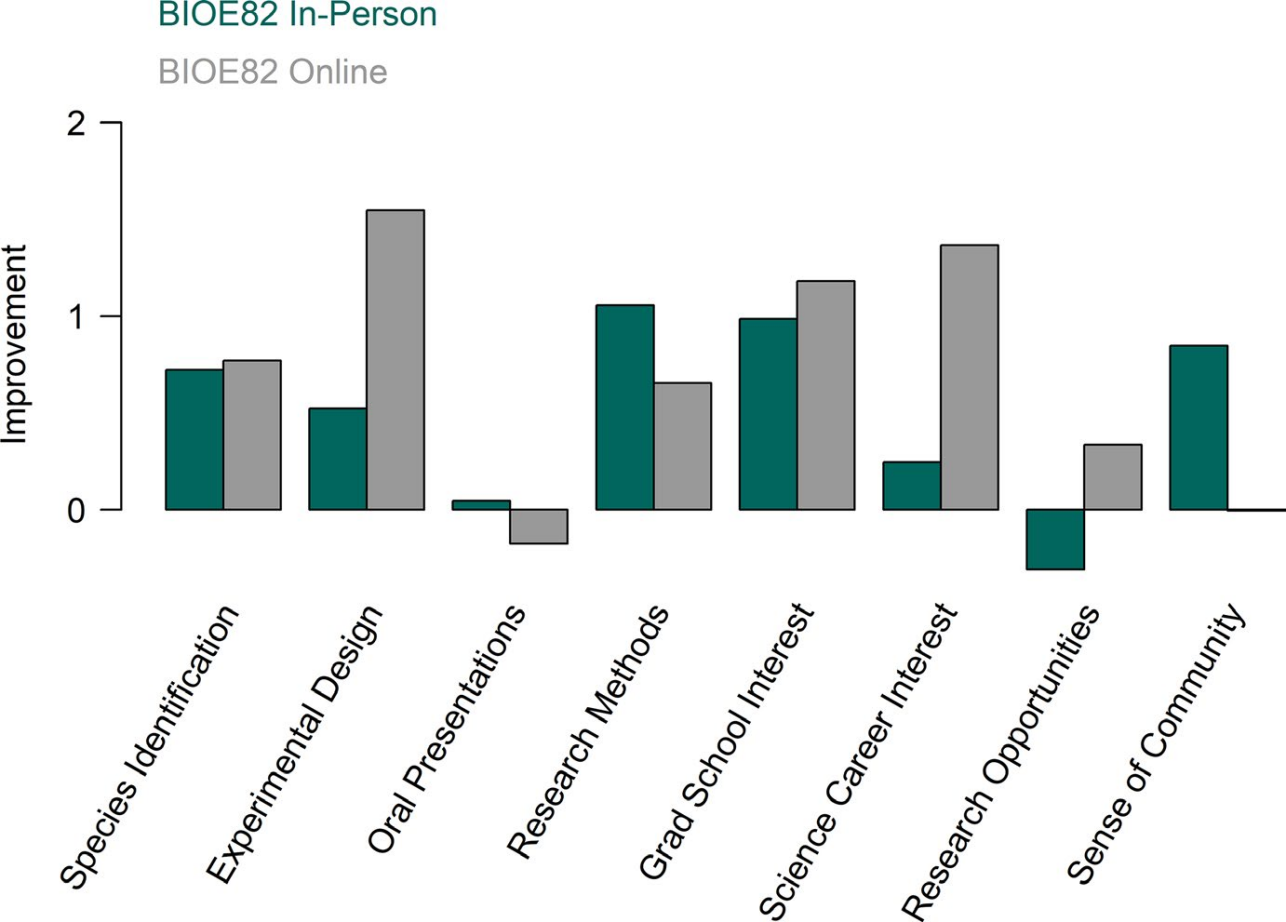
Comparison between improvement values from online and in‐person field course. Improvement values are the difference in self‐ranked confidence between pre‐ and postsurveys, across all demographic groups (in‐person pre = 271, post = 81; online pre = 33, post = 7)

We found that three themes emerged from the data: (a) Community Building, (b) Self‐Efficacy, and (c) Connection to the Field (Figure 1). Below, we explore the effect of the course components on these themes, comparing outcomes for students participating in the in‐person versus the online versions of the course.

### 3.1 Community building

Community, sense of belonging, and connection to classmates are all important factors for building an inclusive space for students to succeed in the sciences (O’Brien et al., 2020). *Intro to Field Research* provides these benefits through intentionally small class sizes (25 students maximum), holistically supported natural experiences, group projects, and multiple field trips designed to ease introduction into the field. When the class transitioned online, the shared field trips could no longer take place.

#### 3.1.1 In‐person field course

Drawing largely from the reflective journals (*n* = 219), we found that students from the in‐person quarters had particular community‐building events that stood out to them. These events, many of which were first‐time experiences, contributed to their appreciation for the course format, and overall sense of community. In one specific instance of this, students in one section of the class swam through a cave to a secluded beach on the other side. Many of the students' fears of partaking in this experience were subdued by their collective engagement. Multiple students reflected on this experience in their journals, including:

Today we shared some pretty insane experiences as a class. It truly is amazing to realize that these types of adventures are possible with this group of people. As we swam through the cave‐tunnel, I heard multiple people saying “this is so out of my comfort zone!” and was awestruck that we were all going through this new experience that may have pushed some people's limits. It definitely brought us closer together and I think that as a group, we become stronger and more empowered.

This conception of overcoming fear persisted throughout the duration of the course, and similar experiences worked to build community among participants. The course structure, including small class size, multiple field trips and small‐group team research projects, also supported the development of collaborative relationships between classmates which improved over the duration of the course. One student reflected on the difference between the first and second overnight field trips:

I'm really glad I took this course in my first quarter because I already started meeting a large group of new people. The small class size allowed for me to get really close and easily make friends. From awkward interactions in Fort Ord *(first trip),* just trying to find a tent, to laughing and enjoying my time with the class. This course really helped me make close connections through our trips and work.

Journal entries like this show how the course supported community building, an important factor in establishing a sense of belonging. Students appreciated the close‐knit relationships they formed through the small class size. They also enjoyed the new friendships they gained through the 7 days total of field experiences provided by the course.

#### 3.1.2 Online field course

When the course transitioned to online, the shared field experiences were no longer possible. Students met weekly over Zoom for 1.5hr, listening to lectures and discussing topics in breakout sessions, and also met when working with a small peer group on a final research project. Drawing from the reflective prompts, open‐ended survey responses, instructor interview, and focus interviews, we found this group project provided the main source of community building for the participants in the online course. While coordinating group work was not always easy, the small‐group connection was important, as one student reflected:

It was okay, communicating when to meet outside of class was a little more difficult. It was nice to be in a small group and have some communication though, so even though timing was difficult to coordinate it was worth it.

Other students felt that the loss of the connection to peers was the biggest downside of the online format, as one student mentioned during a focus group interview when asked what they felt was the biggest thing lost was:

I think one‐on‐one communication between peers. Being able to work together, like “oh I am not really sure what this plant is.” Being able to problem solve as a group. Personally, I like talking and bouncing ideas off people and it's just harder to do through a Zoom format.

Without the shared experiences in the field and the in‐person connections, the online format provided little support for community building. This was supported by the survey data as well (based on an average 33% response rate); the online participants had no change in sense of community (Figure 1).

### 3.2 Self‐efficacy

Self‐efficacy is the confidence that one can successfully complete the behavior required to produce a result (Bandura, 1977). Science self‐efficacy thus can be understood as the conviction that one has to successfully complete tasks required for science, such as research skills. Robnett et al. (2015) argue that science self‐efficacy can be used as a mediator to better understand the positive effects of research experiences. Byars‐Winston et al. (2016) similarly argue that the “self‐efficacy factor” and “science identity factor” can better capture student outcomes in science courses. To understand how self‐efficacy was impacted by *Intro to Field Research*, we looked at how students discussed their experiences in their research projects as well as their perceived skill gains.

#### 3.2.1 In‐person field course

In the in‐person field course, students completed two small‐group, rapid research projects: one on their first overnight field trip and the other on their second overnight field trip. Between the two research experiences, we often saw self‐reported increases in students’ self‐efficacy in completing the research project based on the reflective prompts. Here, one student reflects on their second research project:

I feel much more confident in the data I'm collecting and in creating research questions. It made me really excited to continue doing research and enhancing my field study skills. I entered the class having little to no experience in field study and now I feel much more confident.

The class provided many students with an opportunity to conduct field research for the first time. Our analysis found 27 co‐occurrences of research and first‐time experience, representing approximately 45% of students (based on responses to prompt about research experience). By having two opportunities to complete projects and applying learning from the first project to the second, students' confidence in their ability to conduct research grew. In survey responses (average response rate ~50%), students reported improvement in research methods and experimental design (Figure 1). Working in a group also influences students' self‐efficacy. One student reflects on their first rapid research experience:

Doing the rapid research project was really cool! I definitely felt like working together with a group of students was really valuable in order to share all of our ideas and make sure that the project made sense. At first we had a very shaky hypothesis and were not quite sure how to test the hypothesis. However, through using our resources and communicating our unique thoughts and ideas, we learned a lot.

Survey and journal prompt responses indicate that shared experiences provided important avenues for gaining self‐efficacy. Working together allowed students to share ideas, work collaboratively, and build confidence through working toward a common research goal. While about 22% (*n* = 60) of students mentioned challenges with group work, the majority of students found value in the group research format.

Students also gained confidence in their ability to actively observe nature. In the reflective prompts (average response rate 30%, *n* =219), over half of the students mentioned their increased ability to observe patterns in nature, name species, and be more aware of their surroundings. One student reflected to a prompt:

I’ve always loved observing nature but I think that this class has helped me to learn to do things with my observations, like ask questions and try to find the answers. It's a lot more engaging and active than passively observing.

Learning field skills not only influences students' self‐efficacy in field research, but also their overall confidence and comfort in the field. As one student said, “I felt … in my element in the field.”

#### 3.2.2 Online field course

Due to limited data, we can say less about how self‐efficacy around research was influenced by the online field course. Students reflected mostly positively on their research experience in the reflective prompts, focus group interviews, open‐ended survey responses, and the midquarter evaluation, including one student who mentioned, “I feel more confident in creating experiments.” This is supported in our survey data, with the online course reporting improvement in experimental design and research methods (Figure 1). The experience also allowed students to connect research across space. One student reflecting on her project, “We all saw a lot of lizards at each of our spaces....One of them was in the LA area and then the other person was in the Sacramento area.” This required students to take ownership of their own individual data at their sites. However, while students felt they did a good job, they felt the loss of access to shared experiences in the field, as one student reflected in the focus group interview:

I think with the group I had, we were able to meet outside of class a lot, so we were able to get it done. Obviously, I would have probably preferred doing it, even having less time, in the reserve. That would've been cool.

Similar to the in‐person course, students reported increased self‐efficacy in making observations in nature. The online course required that students make weekly observations of their “Place in Space.” This led to 90% of students (*n* = 10) mentioning, across the above‐mentioned data sources, increased ability to recognize patterns in nature, and be better observers of their space. One student reflected in an open response on the postsurvey:

I found the weekly outings to my place in space helped me learn how to be a better naturalist and observer. At the beginning of this class I took nature for its face value but now I feel like when I visit a space I can visualize deeper interactions that are going on.

Thus, while students in the online class did not have shared field experiences, the process of going outside in their space allowed them to practice observation skills and improve their self‐efficacy in being an active, inquisitive observer of nature.

### 3.3 Connection to the field

A major goal of this course is to help students develop a connection to their local environment and its natural history. Through holistically supported experiences, the course aims to dismantle barriers that students may encounter when experiencing and interacting with nature in ways for the first time. Spaces like this are incredibly important, especially given the historic patterns of exclusion and lack of access to green spaces by URMs (Byrne, 2012). A connection to nature is also known to impact interest in conservation and sustainable behaviors (Nisbet et al., 2009).

#### 3.3.1 In‐person field course

The course has four field trips, which largely utilize the UC Reserve system. The class starts with a day trip to the upper UC Santa Cruz campus reserve, introducing students to the natural history of the campus. The second trip is overnight to a UC Reserve 1 hr away that has unique coastal prairie and shrubland ecosystems. The third trip is a day trip to Año Nuevo Natural Reserve and to local tidepools. The final trip is a 2‐day, overnight trip to the Landels‐Hill Big Creek Reserve in Big Sur. These field trips, each covering unique ecosystems, greatly affected students' connection to the field and the natural environment. As one student reflected during the last trip:

The way that I engage with the field has changed pretty dramatically. I feel like I have a new appreciation for wildlife and the environment, especially after the Big Sur hikes we did. Even though the hikes were exhausting, they were definitely worth it and now I want to protect these areas even more.

This increased appreciation was often mediated through increased field skills and knowledge. The appreciation was strongly reflected in the reflective prompts (*n* = 219), with the code “appreciation” having the highest occurrence. In fact, the code “connection to nature” co‐occurred with “appreciation” nearly 60% of the time. This increased knowledge often led to students craving more information, and a desire to continue their field experiences, as one student wrote:

I feel much more confident exploring the outdoors and I find I'm much more engaged with the natural environment. I'm thinking about taking more field‐based courses in the spring. I also want to get a plant‐guide to bring with me exploring.

This idea of exploration was common in student's reflections. With increased confidence and appreciation, students wanted to take their new‐found skills and further explore their natural environments.

#### 3.3.2 Online field course

For the students and instructors of the online course, the added stress of a global pandemic and a new instructional format shifted outcome expectations. Instead of field trips, students spent time outdoors at their own places and watched videos to learn about the local environment. Based on the focus group interviews, midquarter evaluation, and open‐end survey responses, we found the students were disappointed that they would no longer be able to have the field experiences that they had expected. A student from one focus group interview, when responding to what they felt was the biggest element lost due to the online transition, said:

The trip to the reserve. Even though I felt like from my point of view, I got to experience and learn a lot from my place in space, I think just having the professors there with you, they find something that's cool and they can tell you about it compared to watching a video and then learning about something..but you might not ever see it in your place and space.

Thus, while this student felt they gained a lot from their own place, the loss of the trip to the reserve was a disappointing downside to the online format, largely due to the loss of natural connection and communication. Despite this, students felt that the course was well‐designed and they still had similar field experiences. As one student mentioned in a focus group interview:

I feel like it was done in a really good way where I was still able to get a good experience with going outdoors and doing things that I'd heard of other people doing in their own field experience.

Intentional course design made a difficult situation work, providing students with opportunities to connect to their local place and develop field‐based skills. This student also reflected on another part of the course design saying, “I really liked the plant and animal of the week videos, and then having a quiz right after it.” This student explained this was where she learned the most in the class (as opposed to the lectures), improving her ability to identify plants and animals.

Another added benefit of this course was the time required outdoors. 50% students (*n* = 10) said it was a much‐needed respite from the hours spent inside in front of a computer. Students responding to the survey said, “It was a nice break from my other summer coursework. Getting to sit out in nature for a few hours a week was very relaxing.” and “It provided me with an excuse to get outdoors which was much needed since I had been a bit of a hermit since the pandemic began.” Time in nature has been proven to provide health benefits (Hartig et al., 2011), and this online field course provided students opportunities to relax and recenter during a trying time.

## 4 DISCUSSION

**FIGURE 2:**
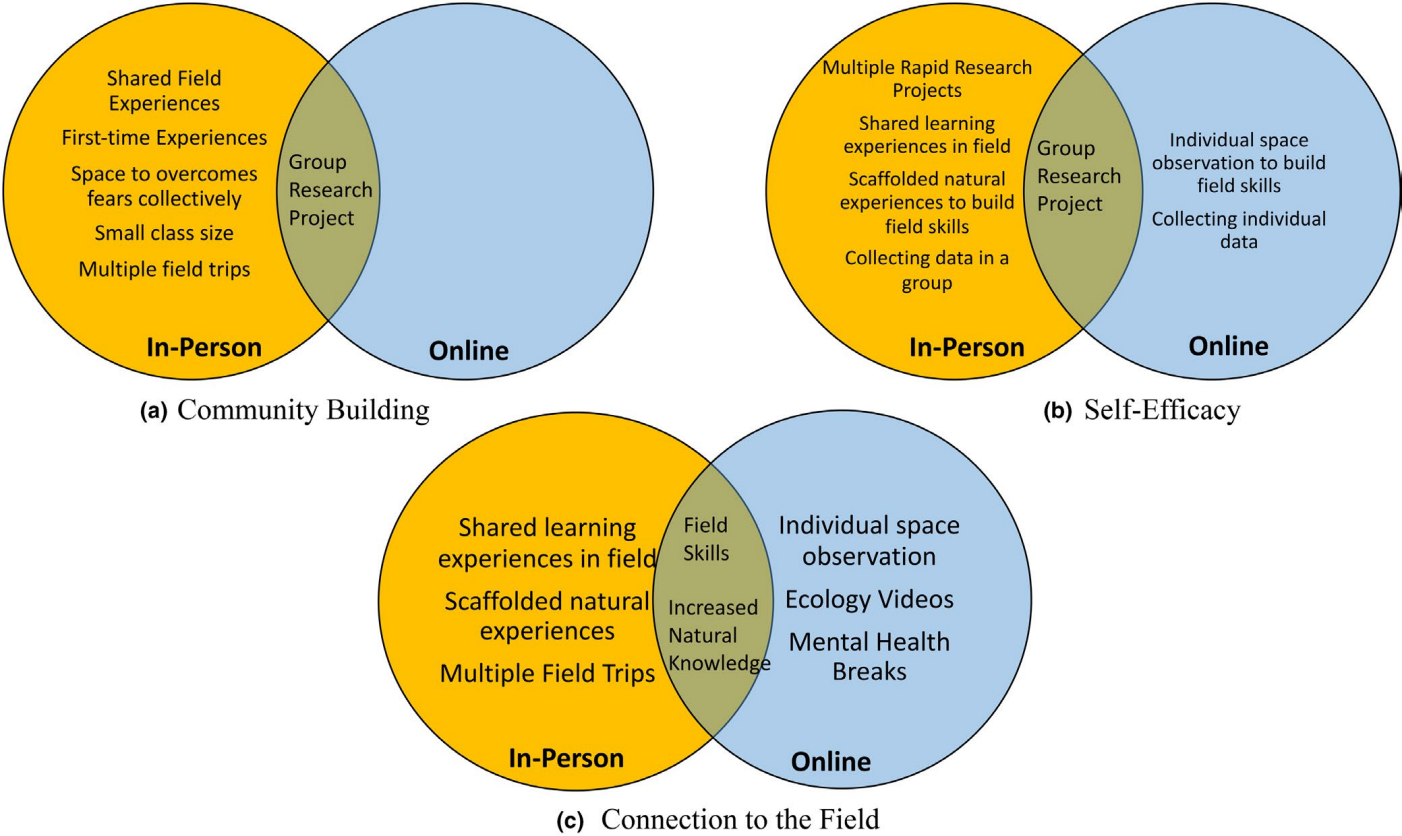
Elements of in‐person and online versions BIOE 82 that contribute to community building, self‐efficacy, and connection to the field

We found that the online version of the course retained many positive factors despite the limitations of an online field‐course format (Figure 2). For example, while we cannot make any causal claims about the impact of the online course on self‐efficacy, we noted increased qualitative expressions of confidence in research and observation skills, which were supported by survey data (Figure 1). The online field course also provided much needed mental and physical health breaks to participants during the global pandemic. Additionally, students enjoyed the time spent in their local space, the course providing them with the skills and knowledge to better recognize local plants and animals, as one student said, “learning how much diversity is all around‐‐even in my own backyard!” The online videos of local environments and flora/fauna were also appreciated by students. They reported they felt the videos, and following quizzes, supported their ability to identify flora and fauna. Interestingly, students in the online course actually had a slightly higher gain reported in species identification self‐efficacy than the in‐person class. We hypothesized that this could be because time devoted to flora and fauna identification in the in‐person course depended on instructor preference, with less overall focus than in the online course.

Despite the retention of many positive outcomes, transitioning online involved some losses. The most notable such loss was the reduced opportunity for community building without shared field experiences and with the limitations of Zoom. With the lack of ability to provide holistically supported natural experiences, accessibility challenges also arose in the online setting. As one student reflected, “So, personally I didn't experience any barriers and I know I'm really really lucky. I have a big wilderness area two blocks away.” This might not have been the case for other students. Other issues, such as an increased responsibility of family care while at home, might have impacted students’ ability to complete assignments and field observations. Another constraint was the online summer offering of this class was only 5 weeks compared to ten. This led to the removal of the CV and cover letter assignments, which comprised a major course element aimed at building science networking and opportunity‐seeking skills. This may have also dampened the connections between classmates and impacted students’ ability and/or interest to find field‐based internships in the future.

We recognize that our study had multiple limitations, such as small sizes for certain data types and quarters, lack of longitudinal data, and limitations to the scope of inference. In future studies, more data collection will help to determine the short and long‐term impacts of both in‐person and online field‐ courses.

As the global pandemic continues to impact higher education, with many universities continuing online instruction in the 2020–2021 school year, it is clear that with intentional design, online field courses can provide benefits to participants. We recommend that instructors take the following in mind when designing online field courses:

1. Take time to cultivate activities for community building (Donovan, 2015). Ideas include organizing fun class activities like a flora and fauna identification competition, book club or personal storytelling curriculum, and coordinating socially distanced hikes for students in nearby areas.
2. Maintain some type of field component, as remote does not mean at a computer. Avoid strictly using videos or previously collected data to connect students to the field and provide opportunities to observe, record and collect data (Dolphin et al., 2019).
3. Include a field‐based research project that allows students to test hypotheses and use data to draw conclusions, experiencing the scientific process (Thompson, 2016). A group project can better support community building (Brown, 2001), such as one in which students in different locations collect a common set of measurements on trees, insects or habitats and work together to investigate broad patterns.
4. Minimize synchronous lecturing. Use Zoom class time for group discussions and sharing, with short, prerecorded lectures viewed individually (Peterson et al., 2018; Young et al., 2020).
5. Give students opportunities to present their research, in groups if possible; the experience of polishing and sharing science can grow confidence as well as sense of community (Mader et al., 2017; Seymour et al., 2004).

While we recognize that shared field experiences are a central driver of the impact of field courses, we hypothesize that by focusing on the key elements of community development, local field engagement, a group field‐based research project, limited synchronous lecturing, and opportunities to present research, participants in online field courses can have similar outcomes to in‐person participants.

The COVID‐19 pandemic has disrupted much of what was considered “normal approaches” to field‐based learning. Through this comparative study, we aimed to clarify the impact of the shift to an online format on student field‐course experiences. While community building declined in the online version of the course, other important dimensions of field‐course experiences remained and contributed to positive outdoor and research experiences. These findings were made possible by our qualitative approach, which gave a voice to the student experience in a more reflective and detailed way than that provided by only quantitative survey scores. Student voice provides context and greater insight into the experiences that shape and drive changes in outcomes. By using a mixed method approach, relying on our quantitative data to triangulate our qualitative findings, we can center our analysis on student descriptions of their experiences, which provides us insight into the “how” and “why” often missing in quantitative data. We encourage more studies of science education focusing on participant outcome to incorporate more student voice in findings, especially for racially marginalized students. We hope these findings can inform iterative design to not only improve online field courses, but also revisit and reevaluate in‐person field‐course structures to increase accessibility (Bennett & Lamb, 2016; Sugerman, 2001), refocus on equitable pedagogy (Warner & Dillenschneider, 2019), and reimagine a field for all.

## CONFLICT OF INTEREST

None declared.

## AUTHOR CONTRIBUTIONS

**Alexandra I. Race:** Conceptualization (equal); data curation (equal); formal analysis (equal); investigation (equal); methodology (equal); project administration (equal); writing–original draft (equal); writing–review and editing (equal). **Maria De Jesus :** Data curation (equal); formal analysis (equal); investigation (equal); methodology (equal); writing–original draft (equal); writing–review and editing (supporting). **Roxanne S. Beltran:** Conceptualization (equal); data curation (equal); formal analysis (equal); funding acquisition (equal); investigation (equal); methodology (equal); project administration (equal); visualization (equal); writing–review and editing (equal). **Erika S. Zavaleta:** Conceptualization (equal); funding acquisition (equal); methodology (equal); project administration (equal); writing–review and editing (equal).

### OPEN RESEARCH BADGES

This article has been awarded <Open Materials, Open Data> Badges. All materials and data are publicly accessible via the Open Science Framework at https://doi.org/10.7291/D12957.

## Data Availability

Aggregate data are available in the publicly accessible repository Dryad at: https://doi.org/10.7291/D12957.

## APPENDIX 1

**TABLE A1:** Description of in‐person and online course formats and components

	In‐Person	Online
Number of Field Trips &/or Time Spent in Field	Four‐weekend field trips (2‐day trips, two overnight trips) and four individual observations	2 hr per week “Place‐in‐Space” Activity
Course Assignments	Field Journal, Reading Summaries, CV/Cover Letter	Field Journal, Reading Summaries (opt. Su20), CV/Cover Letter (S20); Plant and Animal of the Week videos/quizzes; Flora/Fauna ID activities (8 major families/orders); iNaturalist activities (S20); Natural History Final Exam (S20)
Research Project Description	Two Rapid Research projects done in groups of 3–4 at Reserves during field trips; Students given apx. 3 hr to develop hypothesis, gather data, create poster and then present to group	Group Research Project assigned based on access to similar data sources; In groups of 3 students had 1–2 weeks to gather data individually at their sites, meet with their groups via Zoom to analyze data and create a presentation, and the record a video of their presentation
Lectures: Time and topics	8 Lectures/2 hr each. Focus on natural history, preparation for field trips, addressing inequity and sexism in the field and careers in science (guest presenters)	10(5) Lectures/1.5 hr each. Focus on natural history, sharing experiences, group discussion, and course requirements

**TABLE A2:** Demographics of the BIOE 82 course at UC Santa Cruz

Quarter	Total # of Students	Number of Sections	FirstGen%	EOP%	%URM
In‐person S19	45	2	47%	36%	38%
In‐person F19	71	3	27%	39%	38%
In‐person W20	44	2	32%	30%	26%
Online S20	26	2	46%	58%	62%
Online Su20	15	1	20%	20%	27%

**TABLE A3:** Count of responses to reflective journal prompts each quarter

Quarter	Total # of students	P1	P2	P3	P4	Total	Average response rate	Handwritten or online?
In‐person S19	45		4	4	6	14	10%	Online
In‐person F19	71	53	49	42	34	178	65%	Handwritten
In‐person W20	44	11	7	2	7	27	16%	Online
Online S20	26	6			2	8	16%	Online
Online Su20	15	3			2	5	17%	Online

**TABLE A4:** Reflective Journal Prompts

Prompt	In‐person	Online
1	1. Has this trip changed the way that you view the UCSC campus? 2. Describe a skill you learned on this trip and how you think you will use it in the future.	1. Have the activities in this class changed the way you observe your place? 2. Do you feel prepared to take this course in an online format? Have you received the support you need from the instructors, your classmates, etc.?
2	1. Did you do anything on this trip for the first time? What was it like? How do you feel now after the experience? a. If you did not experience anything for the first time, please describe a meaningful experience you had on the trip.	
3	1. Reflect on the connections you have made thus far in the class. Have your connections with your classmates changed since your shared experience at Fort Ord? 2. Describe an experience you shared with a classmate on the trip to Ano Nuevo.	
4	1. Has the way you engage with the “field” changed over the course of this program? 2. How did this overnight trip compare to your first overnight trip?	1. Have you made a connection with a classmate? 2. How has your research project experience been?

**TABLE A5:** Summary of data collected

	Pre/Post surveys	Reflective journal prompts	Focus group	Instructor interviews	Informal course evaluation	Case study
In‐person S19	✓	✓	✓			
In‐person F19	✓	✓	✓			✓
In‐person W20	✓	✓		✓		
Online S20	✓		✓		✓	
Online Su20	✓	✓	✓	✓		✓

**TABLE A6:** Summary of data response rates

	Spring 2019	Fall 2019	Winter 2020	Spring 2020	Summer 2020
Total # of students	45	71	44	26	15
Format	In‐person	In‐person	In‐person	Online	Online
Pre‐surveys collected (#/Response Rate)	41 (91%)	70 (99%)	44 (100%)	12 (46%)	9 (60%)
Post‐surveys collected (#/Response Rate)	26 (58%)	43 (61%)	12 (27%)	12 (46%)	3 (20%)
Focus group # of participants	9	5	0	1	1
Mid‐quarter course eval	–	–	–	9	–
Case study	–	23	–	–	15
